# *In vitro* and *in vivo* modeling systems of supratentorial ependymomas

**DOI:** 10.3389/fonc.2024.1360358

**Published:** 2024-02-26

**Authors:** Emily A. Hatanaka, Joshua J. Breunig

**Affiliations:** ^1^ Board of Governors Regenerative Medicine Institute, Cedars-Sinai Medical Center, Los Angeles, CA, United States; ^2^ Department of Biomedical Sciences, Cedars-Sinai Medical Center, Los Angeles, CA, United States; ^3^ Center for Neural Sciences in Medicine, Cedars-Sinai Medical Center, Los Angeles, CA, United States; ^4^ Samuel Oschin Comprehensive Cancer Institute, Cedars-Sinai Medical Center, Los Angeles, CA, United States; ^5^ Department of Medicine, David Geffen School of Medicine, University of California, Los Angeles, Los Angeles, CA, United States

**Keywords:** glioma, oncofusion proteins, rare cancer, tumor modeling, pediatric tumor, brain cancer, CNS tumor

## Abstract

Ependymomas are rare brain tumors that can occur in both children and adults. Subdivided by the tumors’ initial location, ependymomas develop in the central nervous system in the supratentorial or infratentorial/posterior fossa region, or the spinal cord. Supratentorial ependymomas (ST-EPNs) are predominantly characterized by common driver gene fusions such as *ZFTA* and *YAP1* fusions. Some variants of ST-EPNs carry a high overall survival rate. In poorly responding ST-EPN variants, high levels of inter- and intratumoral heterogeneity, limited therapeutic strategies, and tumor recurrence are among the reasons for poor patient outcomes with other ST-EPN subtypes. Thus, modeling these molecular profiles is key in further studying tumorigenesis. Due to the scarcity of patient samples, the development of preclinical *in vitro* and *in vivo* models that recapitulate patient tumors is imperative when testing therapeutic approaches for this rare cancer. In this review, we will survey ST-EPN modeling systems, addressing the strengths and limitations, application for therapeutic targeting, and current literature findings.

## Introduction

In rare cancers where there is a limited human population for clinical trials, the usage of appropriate preclinical models is generally imperative in the development and testing of therapeutic approaches to advance treatment. This is especially prevalent in brain cancers, with dismal fatality rates and variable resistance to common clinical strategies. Supratentorial ependymomas (ST-EPNs) are a relatively rare subgroup of ependymomas (EPNs). Only a few ST-EPN patient case reports exist, and this clinical subtype has been difficult to diagnose before the routine inclusion of clinical molecular sequencing methods (1). Due to low incidence, clinical management and therapeutic protocols of this disease have often been debated and controversial. In this review, both *in vitro* and *in vivo* models of ST-EPNs will be discussed. Key advantages and limitations will be addressed for each modeling system as well as current advances in the understanding of this rare tumor type.

## Supratentorial ependymomas

EPNs are rare tumors of the central nervous system (CNS), occurring in both pediatric and adult patients. General standard of care for EPN patients includes maximal surgical resection and adjuvant radiotherapy, depending on age and location (2). Chemotherapeutic application has been controversial, and clinical benefits have been questioned largely due to chemoresistance (3, 4). EPNs are stratified into subgroups determined by tumor location: supratentorial (ST), infratentorial/posterior fossa (PF), and spinal ependymoma (SP) (5, 6). Molecular analysis suggests genetic profiles that are distinct to each of these compartments.

ST-EPN tumors are largely classified as either ST-EPN–*ZFTA* fusion-positive (zinc finger translocation associated, previously known as *C110rf95*) or ST-EPN–*YAP1* fusion-positive (yes associated protein 1) (7); classification of these two subgroups are summarized in **Table 1**. The most significant pathogenic fusion *ZFTA*–*RELA* (v-rel avian reticuloendotheliosis viral oncogene homolog A), found in more than 70% of ST-EPN tumors, results from a chromothriptic event on chromosome 11q13.1. Together, the ZFTA–RELA fusion protein activates NF-κB signaling and is highly tumorigenic (8). ST-EPN–*ZFTA* fusion-positive tumors are one of the most aggressive classes of tumors and have a 5-year progression-free survival of <30% (7). Studies have identified non-RELA ZFTA fusion ST-EPN tumors such as ZFTA-MAML2 and ZFTA-NCOA1/2; these are rare cases and have worse patient outcomes (9). The less common driver gene fusion subgroup includes *YAP1*, a transcriptional cofactor regulating proliferation and maintaining stem cells. Known fusion of *YAP1* with the mastermind-like domain-containing protein 1 (MAMLD1) has been shown to disrupt Hippo signaling and promote tumorigenesis (10, 11). ST-EPN–*YAP1* fusion-positive patients have been shown to have a better prognosis with a 5-year progression-free survival of 66% (7). Despite recent work that expands on knowledge of driver-fusions in ST-EPN subgroups, a better understanding of the subgroup characteristics is needed to develop new targeted therapies. This requires proper modeling systems that recapitulate the tumors found in human patients.

**Table 1:** Molecular subgroups of ST-EPN with associated fusions, WHO grading, demographic, and clinical outcome.

Subgroup	Recurrent Fusion	WHO Grade	Age Group	Outcome
**ST-EPN-ZFTA fusion-positive**	ZFTA-RELA*	II/III	Children Adults	Poor
	*ZFTA-YAP1*			
	*ZFTA-MAMLD1*			
	*ZFTA-NCOA1/2*			
**ST-EPN-YAP1 fusion-positive**	YAP1-MAMLD1*	II/III	Children	Good
	*YAP1-FAM118B*			

*ST-EPN fusion better characterized in the literature.

### *In vitro* modeling

*In vitro* models allow for the characterization of tumor cells on transcriptomic, genetic, and epigenomic levels, giving insight into tumor cell progression and potential molecular targets. In addition, they provide a controlled the environment to test pharmacological efficacy and perform drug screenings. Due to limited human tumor samples, preclinical *in vitro* and *in vivo* models of ST-EPNs have been restricted, highlighting the utility of ependymoma cancer cell lines. Prior works have established primary short-term and long-term cell lines derived from ST-EPN patient samples that can be cultured as adherent or neurosphere cultures (12–15). Utilizing these cell lines has uncovered transcriptional signatures and cellular programs that function in tumor aggressiveness (16).

#### 2D adherent cultures

Overcoming past hurdles of short cell lifespans and slow-growing cultures, the successful establishment of ST-EPN permanent cell lines has been reported in the literature. One of the earliest reported lines was published by Yu et al., establishing the ST-EPN *ZFTA*–*RELA* line BXD-1425EPN (12). This line was derived from xenograft EPN tumors that were digested and cultured. Investigators cited the usage of tumor samples that were initially growing *in vivo* in mouse brains to allow for rigorous selection and improve cell culture viability (12). BXD-1425EPN successfully grew as a monolayer culture, could be serially passaged and transplanted orthotopically to form tumors (12). This line is frequently utilized in publications and has aided in understanding the mechanisms of tumorigenesis and therapeutic response. As shown in de Almeida Magalhães et al., investigators describe a mechanism of therapeutic resistance to Hedgehog (Hh) pathway inhibitors, mediated by cilia loss in ST-EPN *ZFTA*–*RELA* cells (16). Here, they observed that recovery of cilia in the tumor cells by treatment with an Aurora kinase A (AURKA) inhibitor improves response to Hh inhibitors promoting tumor cell death (16). These results uncover a targetable pathway of resistance and suggest a novel combinatorial therapeutic approach for EPN.

Since the early advent of available ST-EPN cell lines such as BXD-1425EPN, protocols have been modified to directly culture primary human EPN tumor samples as adherent monolayers without the need for initial xenograft transplants (13, 17).

In addition to primary patient tumor cell lines, other adherent cultures have been used to further understand tumorigenesis. Cell of origin for ST-EPNs has been hypothesized to originate from neural stem cells (NSCs) or radial glia-like cells (18, 19), and a deeper mechanistic understanding of how ST-EPN fusions transform these cells and drive tumor formation can give insight into potential therapeutic targets. Kupp et al. transduced mouse NSCs and human embryonic kidney 293 cells (HEK293T) with ZFTA fusion proteins to investigate transformation (20). In this study, they confirmed the role that *ZFTA* fusions have in nuclear translocation, chromatin modification, and promiscuous expression (20). ZFTA–RELA^FU^ *^S^* proteins were found to translocate to the nucleus and upregulate *ZFTA^FUS^* signature genes such as the EPN oncogene *EPHB2* and zinc finger protein *GLI2* in cultures (20). Through chromatin immunoprecipitation sequencing, ZFTA was found to be responsible for genome binding (20). *ZFTA*–*RELA^FUS^* binding sites included *ZFTA^FUS^* signature genes *EPHB2*, *GLI2*, and *L1CAM (* 20 *).* The *L1CAM* gene has been used as a diagnostic marker for ST-EPN ZFTA fusion-positive tumors (21) and may serve as a therapeutic target to inhibit in future studies. Additionally, the SWI/SNF, SAGA, and NuA4/TIP60 protein complexes responsible for chromatin remodeling and activation were recruited in *ZFTA–RELA^FUS^* cells (20). Together, these data reveal mechanisms of *ZFTA*–*RELA*-dependent transformation and identify therapeutic targets to perturb *ZFTA*–*RELA*-driven transcription to mitigate downstream tumor progression.

#### 3D neurosphere cultures

ST-EPN patient cancer cell lines have been shown to grow reliably in neurosphere cultures—perhaps due to their intrinsic proclivity to form rosette-like structures *in vivo*. Milde et al. generated the neurosphere culture line DKFZ-EP1NS from a patient with WHO grade III ST-EPN and investigated the response to therapeutic agents (14). The DKFZ-EP1NS cells treated with the common chemotherapeutics vincristine, cisplatin, and temozolomide alone in culture did not significantly reduce cell viability measured by metabolic activity (14). However, investigators observed a significant decrease in metabolic activity in ST-EPN neurosphere cultures in response to histone deacetylase inhibitors (HDACi), highlighting the potential use of epigenetic modifiers such as HDACi in ependymoma treatments (14). The utility of HDACs was similarly found in Antonelli et al. (22), where they have been further investigated as potential biomarkers in cancers. One such member *HDAC4* was identified to be overexpressed in ST-EPN–*ZFTA* fusion-positive patient samples and correlated with worse outcomes and low levels of NK cells (23), identifying HDAC4 as a prognostic biomarker and future potential therapeutic target.

3D culturing allows for various benefits including the ability to maintain a self-renewing population of cancer stem cells (CSCs). CSCs have been identified as a source of tumor progression and resistance to treatment, proving to be an attractive model when investigating therapeutic efficacy and drug targeting (13, 14). *ZFTA*–*RELA* fusion tumors have been shown to strongly express the neural stem/progenitor gene nestin, which was associated with worse patient outcomes, suggesting a functional role of stem populations in tumor progression and prognosis (18). Sabnis et al. identified a subpopulation of *BLBP*-expressing CSCs in pediatric EPN patients, that correlated with increased susceptibility to relapse or death (24). In this study, they investigated the ability to target and inhibit *BLBP* by PPAR (peroxisome proliferator-activated receptors) antagonists, utilizing the *BLBP* high expressed ST-EPN neurosphere cell line DKFZ-EP1NS (24). Investigators treated the ST-EPN spheres with PPAR antagonists and observed both a significant reduction of BLBP expression as well as reduced cell viability and migration, highlighting CSCs and its subsets as potential therapeutic targets to reduce tumor invasion and progression (24).

#### *In vitro* limitations

Even though several *in vitro* models have been established to further investigate ST-EPN, there are significant limitations. Long-term culturing and passaging of human cancer cell lines increase the potential for selective and progressive changes both genetically and phenotypically (25, 26). Torsvik et al. identified an accumulation of genetic changes that are indicative of genetic drift in long-term passages of a commonly used glioblastoma (GBM) cell line (27). Investigators observed loss of the typical human GBM DNA copy number profile, identifying gains and losses of loci, causing divergence from the original cell line. In addition, these cultures morphologically differed and were observed to have an increased rate of cell growth both *in vitro and in vivo (* 27 *)*. Together, these observations highlight critical changes in cultured cancer cell lines that may alter results such as response to therapeutic intervention. Another key limitation is that *in vitro* modeling systems do not fully capture the complexity of the tumor microenvironment, such as the high levels of intratumoral heterogeneity (28) and the cell-to-cell interactions in a living system. 3D culture systems have been used to mitigate some of these limitations and better model the tumor vasculature (29, 30); however, there is still a need to model the complete microenvironment including the brain’s immune compartment.

### *In vivo* modeling

*In vitro* models have significant limitations when modeling the complex and heterogeneous tumor microenvironment and natural innate and adaptive immune responses. Due to this, results found in *in vitro* systems have not always translated well to clinical application. In addition, aspects such as how *ZFTA* and *YAP1* fusions function in tumor initiation and formation cannot be fully elucidated with *in vitro* models alone. To address these shortcomings, various *in vivo* models have been established and will be discussed in this section.

#### Patient-derived orthotopic xenograft models

Patient-derived xenograft (PDX) models can be generated from transplanting cultured human tumor spheroids or primary human tumor samples into immunocompromised animal recipients. Directly transplanting primary tumor samples bypasses time in culture, which may aid in preserving components of the tumor and reduce genetic alterations. Heterotopic transplantations are performed via subcutaneous injections of tumor cells. Subcutaneous tumors allow for ease of transplantation and monitoring; however, they lack the original tumor environment. Patient-derived orthotopic xenografts (PDOXs) may serve better for translation applicability due to transplantation in the same region where the sample originated, recapitulating the microenvironment more closely. Regional differences in tumor gene expression have been identified, which highlights the importance of the location of tumor seeding (31, 32).

Recent studies have worked to establish and characterize both PDX and PDOX ST-EPN models in mice (12, 15, 33, 34). Brabetz et al. established a biobank of 30 characterized brain tumor PDOX models in NOD-scid IL2R-gamma (NSG) mice, 3 of which were from EPN patients and 1/3 belonging to the ST-EPN *ZFTA*–*RELA* group (33). Histology of the EPN PDOX models identified pseudo-rosette structures similar to human patient histology (33). Whole-exome and whole-genome sequencing was performed, identifying the presence of *ZFTA*–*RELA* fusion in the PDOX samples, as well as the loss of *CDKN2A/B*, commonly found in the *ZFTA*–*RELA* fusion subgroup (33). In addition, DNA methylation levels were found to be similar between the human tumor samples and PDOX models (33). Together, the robust histological and genetic characterization of the PDOX models confirmed similarity to the original human tumors (33). With stable PDOX lines that maintain key tumoral features *in vivo*, future work in testing therapeutic responses can be more faithfully observed.

A major disadvantage to the PDX/PDOX modeling system includes potential clonal selection *in vivo*, where outgrowth of an aggressive clone can alter tumor progression and potential therapeutic response. An additional disadvantage includes a low percentage of successful engraftment. Brabetz et al. reported orthotopically transplanting 100 patient tumor samples, with only 30 of those successfully engrafted and passaged subsequently, with similar efficiencies observed by others (35).

A final key disadvantage to this modeling system includes the necessity of transplanting human tumor cells into immunocompromised mice. There has been a growing appreciation of the immune composition in the tumor microenvironment in tumorigenesis and response to therapeutic targeting (36, 37). Utilizing mice with severe immunodeficiencies lack integral cellular populations that may influence tumor progression and therapeutic responses.

#### RCAS/tv-a system

Replication-competent ASLV long terminal repeat (LTR) with a splice acceptor (RCAS) vectors are a part of the avian sarcoma leukosis virus A (ASLV) subgroup of retroviruses, utilized for its gene delivery system. The RCAS virus enters cells via the TV-A receptor protein, typically found on avian cells, and incorporates viral DNA into the host genome. Since the TV-A protein is not normally expressed by mammalian cells, it can be cloned into cells under tissue-specific promoters. The RCAS system has been utilized for tumor modeling by introducing oncogenes of interest into cells, driving tumorigenesis *in vivo*.

Prior work has shown expression of the common ST-EPN *ZFTA*–*RELA* fusion-activated NF-κB signaling and transformed mouse embryonic NSCs *ex vivo* (8). Based on this, Ozawa et al. utilized the virus-based RCAS/tv-a system to deliver the human *ZFTA*–*RELA* fusion gene expression into cells in mouse brains to investigate the ability to form an ependymoma (38). Specified cell types such as Nestin+ cells, GFAP+ cells, and BLBP+ cells were targeted by using transgenic tv-a mouse strains. Investigators found expressing the *ZFTA*–*RELA* fusion (*RELA^FUS1^*) *in vivo* via the RCAS system upregulated NF-κB-associated transcriptional programs and drove tumor formation, characteristic of EPNs in humans. In addition, they identified noncanonical NF-κB transcriptional programs in the *ZFTA*–*RELA*-driven tumors, such as dysregulation in genes involved in cell-to-cell adhesion, vesicular transport, and immune processes/inflammation. Similar dysregulation has been observed in non-*ZFTA*–*RELA* EPN tumors as well as other cancers, highlighting the importance of non-NF-κB-related programs in cellular transformation. This set of genes can be further used to investigate new potential targets to abrogate transformation.

One of the limitations of using the RCAS/tv-a system when modeling tumors *in vivo* includes the necessity of a TVA transgenic mouse line, as normal mammalian cells do not express the required TVA receptor that is required for RCAS virus infection. Generation of these transgenic mouse lines can be time-consuming and expensive. Another key limitation of the RCAS/tv-a system includes the limited carrying capacity of the RCAS virus along with intrinsic bottlenecks on infected cell types due to the viral properties and TVA receptor expression *in vivo* (39). This largely can limit the oncogenes that can be studied as well as the potential for successful tumor formation. Lastly, the RCAS/tv-a system relies on the usage of viruses (and often the transplant of 10^5^–10^6^ chicken DF-1 viral propagating cells), which may induce an immune reaction in the host (38, 40, 41). Aberrant immune activity could confound results, especially when observing therapeutic responses, making it difficult to distinguish what is true biology and what is an artifact of viral induction.

#### Transposon-based system

Transposons are genetic elements that shift and integrate their position from one location to another within the genome. Transposable elements can be engineered and utilized for genetic modification such as inactivating or expressing genes of interest (42). Non-autonomous DNA transposons allow for direct insertion of genetic material via transposase activity. Commonly used transposon systems for mammals include Tol2, Sleeping Beauty (SB), and piggyBac (pB) (43). These systems allow for integration of designed genetic material of interest into the host genome.

Pajtler et al. describe the usage of the Tol2 transposon system to mediate gene transfer to model ST-EPN–YAP1 and investigate the role of *YAP1* fusion in tumorigenesis (10). In this study, they developed a human *YAP1*–*MAMLD1*-driven ST-EPN–*YAP1* mouse model, where the fusion was encoded in a pT2K expression plasmid along with Tol2 transposase and luciferase under the constitutively active CAG promoter. This plasmid was then injected into the lateral ventricle of E13.5 embryos, followed by electric pulses delivered by electrode paddles for an *in utero* electroporation. Post electroporation, they observed an increase of luciferase in the brain over time, leading to 100% penetrant tumor formation. In addition to tumor formation, they observed molecular characteristics of the *YAP1*–*MAMLD1*-driven tumor such as high expression of the radial glial neural stem cell marker *PAX6*, suggesting a transforming role of *PAX6*+ cells in ST-EPN–*YAP1*. Investigators identified key molecular mechanisms such as a requirement of both the Hippo pathway regulator *YAP1* and the mastermind-like protein *MAMLD1* for nuclear transport. Downstream of this, *TEAD* and *NFI* binding motifs were found to interact with the *YAP1*–*MAMLD1* fusion, further uncovering this tumorigenic pathway (10). Current work is investigating the potential for the use of inhibitors blocking the interaction of YAP and TEAD which could be a possible target in the ST-EPN-YAP1 subtype (44).

Similarly, Arabzade et al. utilized the pB transposon-based system with *in utero* electroporation to model the *ZFTA–RELA* fusion *in vivo* (45). The authors observed nuclear localization of the *ZFTA*–*RELA* fusion and histological features of ependymoma in mice that succumbed to tumor formation around 60 days post-birth. In this study, they performed ChIP-seq and CUT&RUN on the ZFTA–RELA fusion tumor cells. Through chromatin profiling, they found that the *ZFTA*–*RELA* fusion bound to active oncogenic enhancer and promoter regions such as *Ephb2*, *Ccnd1*, *Akt1*, and *Notch1*, identifying transcriptional programs altered by *ZFTA*–*RELA* fusion (45). Both studies have revealed key molecular functions of ST-EPN fusions and how they function in forming tumors in the brain (44, 45). Moving forward with this, these molecular targets can be further studied as potential therapeutic targets.

DNA transposons have addressed some limitations of viral-based systems; however, this approach has intrinsic drawbacks. A main limitation is that the transposon systems can target minimal consensus sites across the genome and will continue to “hop in and hop out” of the genome in the presence of the transposase, causing insertional mutagenesis, inconsistencies in phenotypes, and other related issues. In addition to the variability in transgene copy number, the transgene load can range from highly supraphysiological to minimal and silenced by epigenetic modifiers (46). However, given the “goldilocks” zone of expression found in other oncofusion-driven tumors (47), this may be advantageous in certain circumstances, as tumors would presumably only be selected for in the appropriate expression range. This, however, remains to be empirically determined.

#### Cre-LoxP Systems-Germline and Somatic

The Cre–LoxP system has been widely used for its utility in genetic editing. This system works by which Cre recombinase recognizes and recombines with a pair of loxP sites located in the genome. Cre then excises the DNA fragment flanked between the loxP sequences, allowing for site-specific gene excision and inactivation (48). Tissue/cell-specific Cre–LoxP excision can be achieved by producing a Cre-driver strain expressed under promoter regions of interest (49). Temporal excision can be achieved by using the tamoxifen- or tetracycline-inducible Cre system. The tamoxifen-inducible Cre system is achieved with a modified Cre that is fused with the estrogen receptor (CreERT) and binds to HSP90 in the cytoplasm. Upon the presence of tamoxifen, CreERT unbinds from HSP90 and allows for nuclear translocation, where Cre–LoxP excision can occur (50). The tetracycline-inducible Cre system can be amenable to a Tet-on or Tet-off system, where Cre expression is either activated or inactivated in the presence of tetracycline.

Kim et al. established the mosaic analysis with dual recombinase-mediated cassette exchange (MADR) method, which uses mice engineered for Cre-LoxP (51). However, this novel, electroporation-based somatic transgenesis method, is used to introduce single-copy gain of function and loss of function oncogenes into neural progenitor cells (NPCs) *in vivo* in mTmG (membrane-targeted tdTomato/membrane-targeted EGFP) heterozygous mice. MADR is incorporated into the cells by insertion of DNA transgene cassettes into the Rosa26 locus, flanked by both the loxP and Flp recombinase target (FRT) sites. In the presence of Cre and Flp recombinases, dual recombination can occur, where the transgene cassette is inserted and expressed under the CAG promoter, leading to constitutive expression of that element. The population of NPCs are targeted on postnatal days 1–2 of the mTmG heterozygous mice by injection of the plasmid mix into the ventricular zone (VZ) and electroporated by swiping electrode paddles across the head of the mouse pup. This system allows for the expression of a milieu of genetic material such as the human driver fusion proteins *YAP1*–*MAML1D* and *ZFTA*–*RELA*. Expression of these common ST-EPN driver fusions leads to the formation of tumors *in vivo* with similar morphological characteristics such as rosette-like structures, defined tumor margins, and lack of invading cells (51).

The MADR system overcomes some disadvantages of viral- and transposon-based systems such as control of site-specific and copy number expression. It is highly amenable to model an endless list of oncogenes and gene fusions of interest in either cell or mouse lines. Limitations to this system include the requirement of mouse strains engineered with the loxP and Flp dual recombinase recognition sites. In addition, in its current configuration, transgenes are not under the control of the natural *cis*-regulatory element and instead use strong artificial promoters, such as the CAG promoter. Usage of these promoters has been used to efficiently drive the expression of transgenes of interest; however, they do not necessarily model expression in a natural biological manner. This limitation can be addressed through the usage of other genetic tools such as CRISPR/Cas9, base editors, and prime editors that may allow for increased specificity in genome manipulation (52, 53).

#### CRISPR/Cas9

As mentioned, there has been work to model ST-EPN tumors by forced expression of the human gene fusions in mouse models; however, an aspect that has not been fully modeled is the gene rearrangement caused by chromothriptic events observed in ST-EPN. This is addressed by Takadera et al., where they used the CRISPR/Cas9 genome editing tool to model the *ZFTA*–*RELA* gene rearrangement (54). The CRISPR/Cas9 system uses a single-guide RNA (sgRNA) to guide the Cas9 endonuclease to a specified region, making DNA double-strand breaks and modifications to the host genome. In this study, they generated sgRNAs to reproduce the *RELA^FUS1^* rearrangement consisting of the first two exons of the mouse homolog of *ZFTA* and exons 2–11 of *RELA*. Using the lentiviral gene delivery system, they injected Nestin-Cre+/−, cag-Cas9+/+ neonatal pups with the *mRELA^FU^* ^S^ vector encoding the sgRNAs. Two months after the lentivirus injection, tumor formation was observed. However, tumor incidence was observed less in the *mRELA^FUS^* mice compared to mice with overexpression of human *RELA^FUS^*. Using the CRISPR/Cas9 system, investigators were able to confirm and model the tumorigenic potential of rearrangement of the *ZFTA* and *RELA* genes. Aspects of chromothripsis such as DNA repair mechanisms of double-strand breaks *in vivo* can be recapitulated using CRISPR/Cas9 technology, highlighting its utility when modeling ST-EPN gene fusions. Better modeling of the gene rearrangement in ST-EPN can reveal neighboring genes and genomic regions that may be altered and or influencing tumorigenesis. A main limitation of this system includes potential off-target effects, where mutations can occur at undesired sites in the genome (55, 56). Additionally, expression of Cas9, a protein derived from bacterium like *Staphylococcus aureus* or *Streptococcus pyogenes*, induces an unintended immune response (57, 58).

## Conclusion

**Figure 1:**
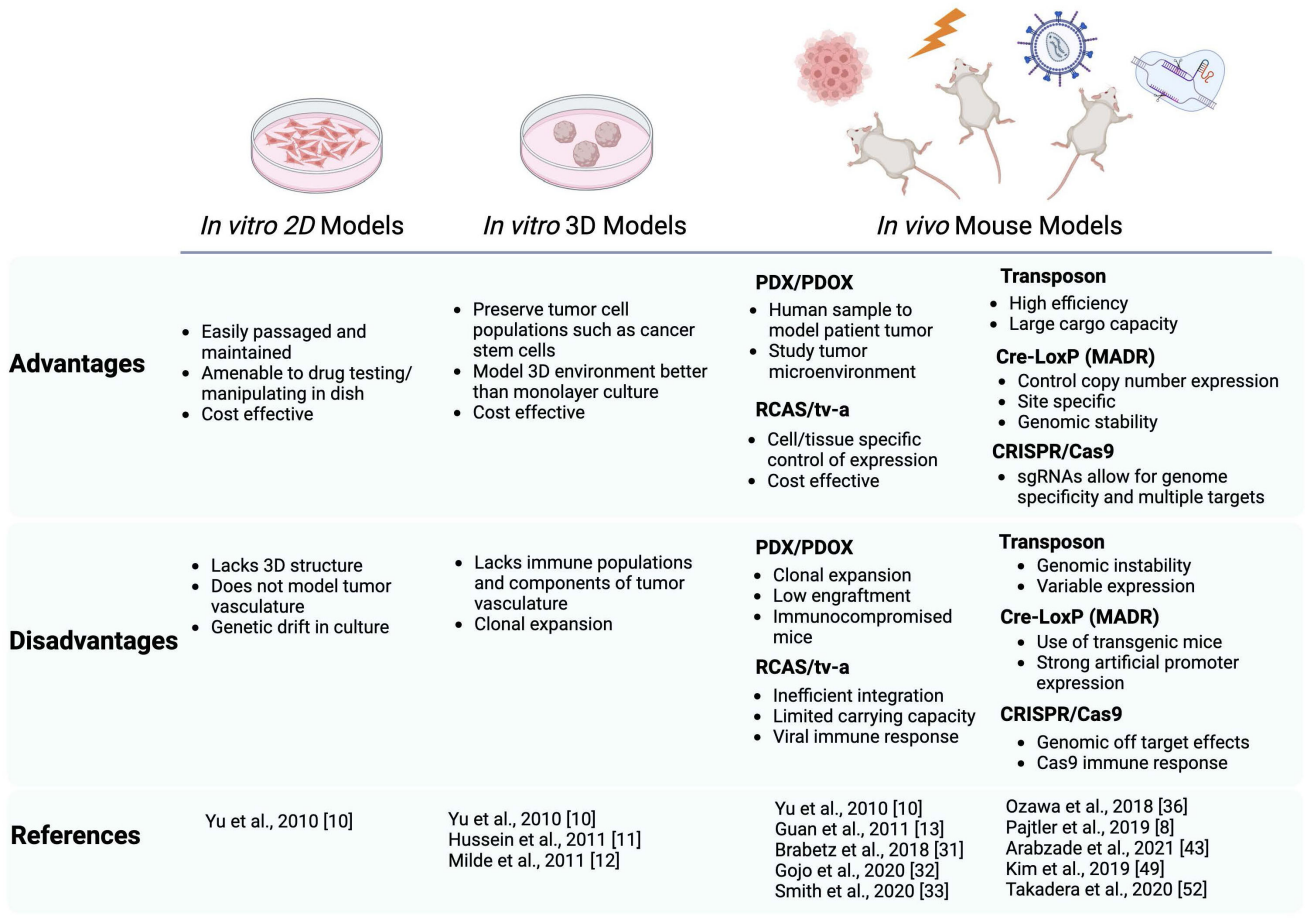
Schematic summarizing the advantages and disadvantages of the current preclinical modeling systems of ST-EPN. Both *in vitro *and *in vivo* models are described with the references utilizing these models cited in this review. In vitro models include 2D adherent cultures and 3D neurospheres, *in vivo* models include PDX/PDOX, RCAS/tv-a system, Transposon based system, Cre-LoxP based (MADR) system, and CRISPR/Cas9. Schematic generated using BioRender.

ST-EPNs can be highly aggressive tumors, particularly in recurrent forms. Moreover, their relative rarity and fundamental biology impose challenges with the generation of *in vitro* and *in vivo* models. As we have discussed, advances in cell culture, transplantation, and mouse somatic transgenesis have quickly enabled the generation of a new cohort of ST-EPN modeling systems, summarized in **Figure 1**. The advent of these models has provided insight into the oncogenic fusions characteristic of ST-EPN and how it is driving tumor formation. In this review, only common gene fusions, *ZFTA*–*RELA* and *YAP1*–*MAMLD1*, characteristic of ST-EPN have been discussed. Additional studies have identified less common *ZFTA*–*RELA*-negative ST-EPN tumors that display fusions such as *EP300-BCORL1* or *FOXO1-STK24* (59). Future investigations are necessary to identify the clinical relevance of these fusions (and other unmodeled ZFTA-fusion subtypes) and implications when classifying ST-EPN tumors. Recent advances in deep sequencing, single-cell RNA sequencing, and other multi-omic technologies utilized in studies cited here, have shed light on the DNA and RNA level, giving insight into potential ST-EPN biomarkers and targets. From these, new insights and disease mechanisms have been elucidated from these systems. Given the acceleration in our knowledge of ST-EPNs and these new models, there is great promise that a new generation of targeted therapeutics and/or combination therapies will emerge.

## Author contributions

EH: Writing – original draft, Writing – review & editing. JB: Writing – original draft, Writing – review & editing.
